# Case report: Delayed outflow obstruction of a DVA: A rare complication of brainstem cavernoma surgery

**DOI:** 10.3389/fneur.2023.1073366

**Published:** 2023-03-14

**Authors:** Kevin Agyemang, Rony Gómez Rodríguez, Victor Hugo Rocha Marussi, Sally Allinson Marte Arias, Alexander Feliciano Vilcahuaman Paitań, José Maria Campos Filho, Feres Chaddad-Neto

**Affiliations:** ^1^Department of Neurology and Neurosurgery, Universidade Federal de São Paulo, São Paulo, Brazil; ^2^Department of Neurosurgery, Beneficência Portuguesa Hospital, São Paulo, Brazil; ^3^School of Medicine, University of Glasgow, Glasgow, United Kingdom; ^4^Department of Neuroradiology, Beneficência Portuguesa Hospital, São Paulo, Brazil; ^5^Department of Neurology, Hospital del Mar, Barcelona, Spain

**Keywords:** cavernous malformation, cavernous angioma, developmental venous anomaly, brainstem, spontaneous occlusion, case report

## Abstract

**Introduction:**

Developmental venous anomalies (DVAs) are considered variants of normal transmedullary veins. Their association with cavernous malformations is reported to increase the risk of hemorrhage. Expert consensus recommends meticulous planning with MR imaging, use of anatomical “safe zones”, intraoperative monitoring of long tracts and cranial nerve nuclei, and preservation of the DVA as key to avoiding complications in brainstem cavernoma microsurgery. Symptomatic outflow restriction of DVA is rare, with the few reported cases in the literature restricted to DVAs in the supratentorial compartment.

**Case:**

We present a case report of the resection of a pontine cavernoma complicated by delayed outflow obstruction of the associated DVA. A female patient in her 20's presented with progressive left-sided hemisensory disturbance and mild hemiparesis. MRI revealed two pontine cavernomas associated with interconnected DVA and hematoma. The symptomatic cavernoma was resected *via* the infrafacial corridor. Despite the preservation of the DVA, the patient developed delayed deterioration secondary to venous hemorrhagic infarction. We discuss the imaging and surgical anatomy pertinent to brainstem cavernoma surgery, as well as the literature exploring the management of symptomatic infratentorial DVA occlusion.

**Conclusion:**

Delayed symptomatic pontine venous congestive edema is extremely rare following cavernoma surgery. DVA outflow restriction from a post-operative cavity, intraoperative manipulation, and intrinsic hypercoagulability from COVID-10 infection are potential pathophysiological factors. Improved knowledge of DVAs, brainstem venous anatomy, and “safe entry zones” will further elucidate the etiology of and the efficacious treatment for this complication.

## 1. Introduction

Cerebral cavernous malformation (CCM) or cavernoma (CM) is a common intracranial vascular lesion, with a prevalence of 0.4–0.9% and an annual hemorrhage rate of 0.5–10% per year (1–3). Approximately one in five cavernomas occur in the brainstem, where symptomatic bleeds are slightly more frequent (3, 4). A pooled analysis found that a history of hemorrhage is the only significant risk factor for further bleeds which, in the brainstem, can lead to severe disability or death (4).

The management of brainstem cavernomas (BSCMs) remains controversial; a recent meta-analysis showed no superiority between microsurgery and stereotactic radiosurgery (SRS) in preventing neurological deficits or further hemorrhage in patients with brainstem cavernomas (5). Microsurgery offers the prospect of immediate remedy from the mass effect and provides protection against future bleeding, but only when complete excision of the BSCM is achieved with no additional deficits.

Developmental venous anomalies (DVAs) are found in 0.14–0.7% of the population on imaging (6, 7) and coexist with a CCM in 25% of cases (8, 9). Porter et al. reported a higher prevalence of venous anomalies at CCM surgery than was identified on MRI or digital subtraction angiography (DSA) pre-operatively (10). Modern high-resolution MR imaging studies support this finding but have found a typical or classic DVA to only be present in one-third of CCMs (11). This association was previously reported to predispose CCMs to hemorrhage (9), but a recent large study concluded that the presence of a DVA added no independent prognostic information (12).

Outflow restriction of a DVA, from stenosis or thrombosis, can result in venous infarction or hemorrhage. Spontaneous thrombosis is rare, with <40 reported cases (<10 infratentorial) in the literature (13–15). Anecdotally, the preservation of all anomalous veins in the region of a CCM is considered an important strategy to avoid additional neurological deficits (16). However, there are no consistent predictors of when DVAs associated with CCMs can be safely occluded without complications (17).

Good anatomical knowledge of the brainstem entry zones (EZs) remains key to avoiding injury to any associated DVA during surgery (18). To the best of our knowledge, there are no reports of delayed symptomatic venous infarction from stenosis of a DVA following resection of a BSCM. We present a case of a pontine cavernoma resection with associated DVA, describe the two brainstem EZs used, and outline our management of this delayed complication.

## 2. Case description

### 2.1. Patient information

The patient was female, in her 20s, with no comorbidities or family history of significance and no regular medications. She presented with a 3-week history of subacute headaches and progressive left-sided hemisensory disturbance.

The clinical examination revealed the patient to be fully conscious, GCS 15, with hypertonia and grade 4+ power on left upper and lower limb examination. Reduced pain, light touch, and vibration sensation on the left were noted. Cranial nerve and cerebellar examinations were unremarkable.

### 2.2. Diagnostics assessment

**Figure 1:**
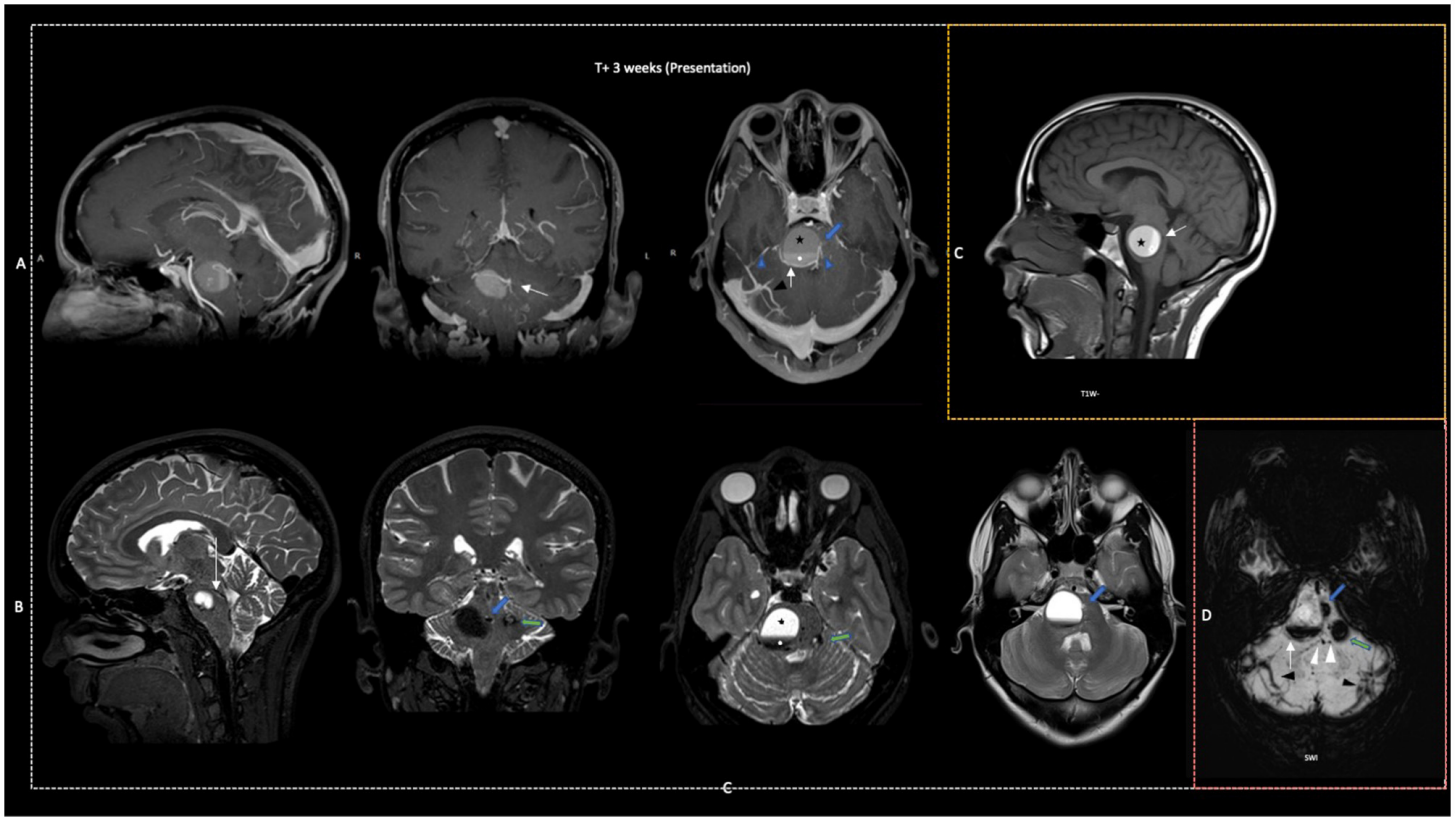
Pre-operative (first surgery). Sagittal, coronal, and axial MRI: **(A)** T1W + Gad; **(B)** T2W; sagittal only: **(C)** TW1 and axial only: **(D)** SWI. Showing partly hyperacute hematoma (black star) of T1W isointense and T2W hyperintense signal. Early subacute hematoma portion (white star) of T1W hyperintense and T2W hypointense signal. Associated: Two CCMs (target/culprit—blue arrow; incidental contralateral cerebellar peduncle—green arrow) seen as blooming artifacts on SWI, popcorn appearance of mixed signal intensity on T2W, outline against the medial aspect of hematoma cavity on T1W-DVA with a retrospectively determined smaller caliber of its main draining vein (white arrow) seen on T1W + Gad filling, serpiginous flow void on T2W and SWI. Draining *via* multiple collector veins on T1W+ (blue arrowheads) into caput medusae appearance of transcerebellar veins on SWI (black arrowhead). Prominent channels can be seen connecting both CCMs likely due to high pressures (white arrowheads on SWI).

MRI brain (Figures 1A–D) showed a cystic lesion in the pons extending to the right middle cerebellar peduncle; a hyperintense portion on T2-weighted imaging (T2W) with the corresponding isointense signal on T1; and T2 hypointense T1 hyperintense part. Susceptibility-weighted imaging (SWI) demonstrated blooming artifacts at the medial aspect of the cyst and the left middle cerebellar peduncle (MCP), along with a caput medusa appearance of cerebellar medullary veins, suggestive of cavernous malformations with associated DVA. The signal characteristics of the cavity were consistent with two stages of hemorrhage (hyperacute and early acute).

Gadolinium-enhanced T1-weighted images (T1W+) showed filling of the cerebellar DVA, with connections to both CCMs and the hematoma cavity. The connecting channels were more prominent on SWI compared to later scans, and we retrospectively interpreted this as due to high pressure in the system. A prominent transmedullary vein can be seen draining *via* a collector vein into the petrosal system. The DVA was visible in the late arterial and the venous phase on DSA with no other vascular malformations identified (Supplementary Figure 1).

Routine hematological and biochemical laboratory tests were normal; the thrombophilia screen was negative. Genetic tests for CCM genes were not performed. The differential diagnosis included neoplasm, arteriovenous malformation, CCM, or DVA. The clinic-radiological picture was in keeping with a symptomatic hemorrhage from the brainstem cavernoma and associated DVA.

### 2.3. Therapeutic intervention

Concerns over the progressive clinical deterioration and extent of radiological compression favored intervention over conservative management. Radiosurgery was contraindicated, given symptomatic brainstem compression. Microsurgery aimed at resecting the cavernoma alone, decompressing the cyst, and preventing future hemorrhage was recommended. High-dose dexamethasone was commenced a week before surgery.

**Figure 2:**
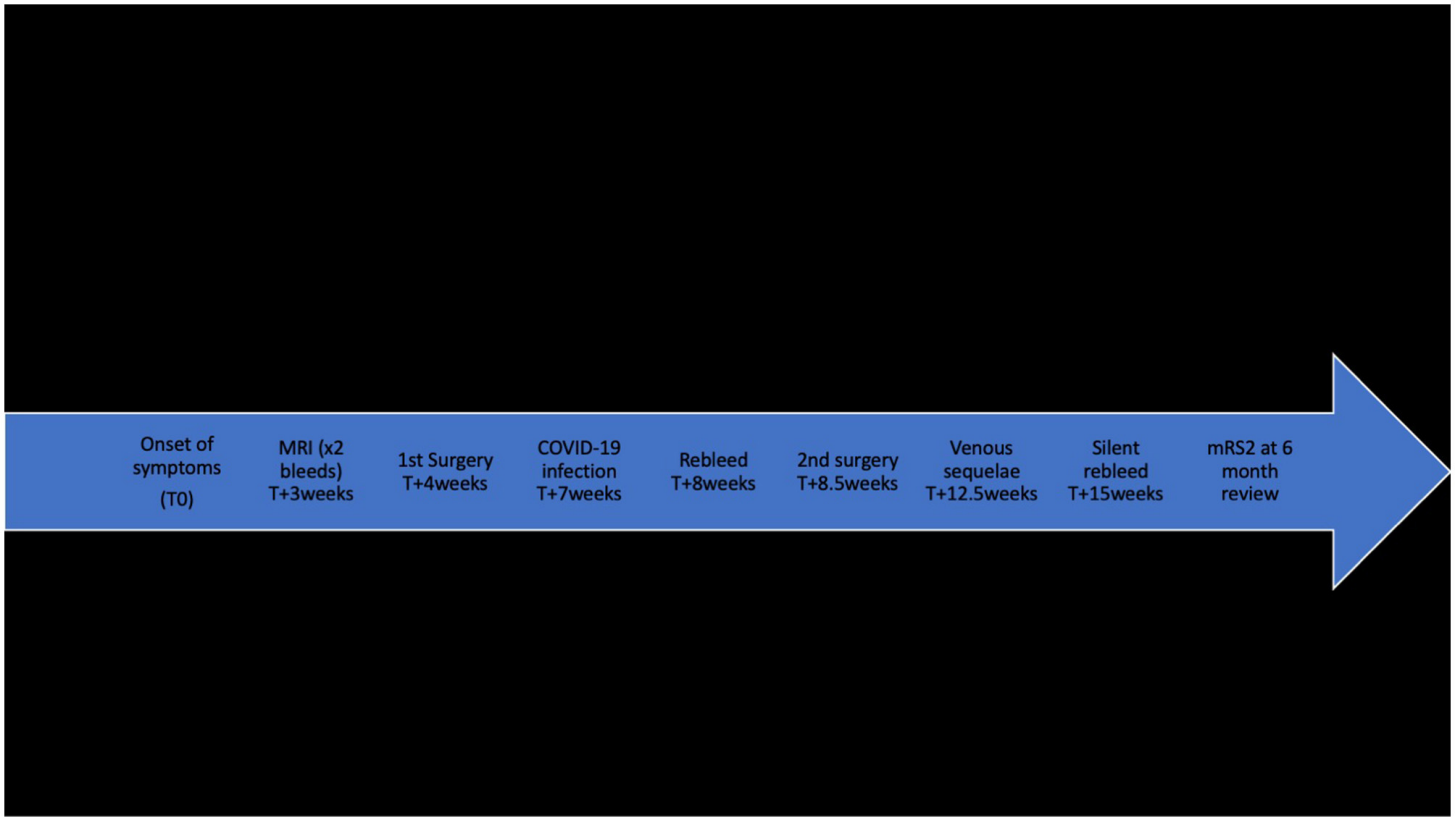
Timeline of clinical course.

A suboccipital craniotomy with peritrigeminal “safe entry zone” to the cyst and cavernoma was chosen, over corridors in the floor of the IV ventricle, to limit manipulation of the dorsally located DVA. The liquefied hematoma was evacuated, and the brainstem was decompressed at the first surgical attempt. The resection of the cavernoma was limited by an inadequate view of its medial position relative to the surgical axis and a posteriorly located superior petrosal vein. Supplementary Figure 2 is an illustration of the location of the CCM and DVA in the brainstem. The surgical Supplementary Video shows the limitation of this EZ. A timeline of the clinical episode is presented in Figure 2.

### 2.4. Follow-up and outcomes

**Figure 3:**
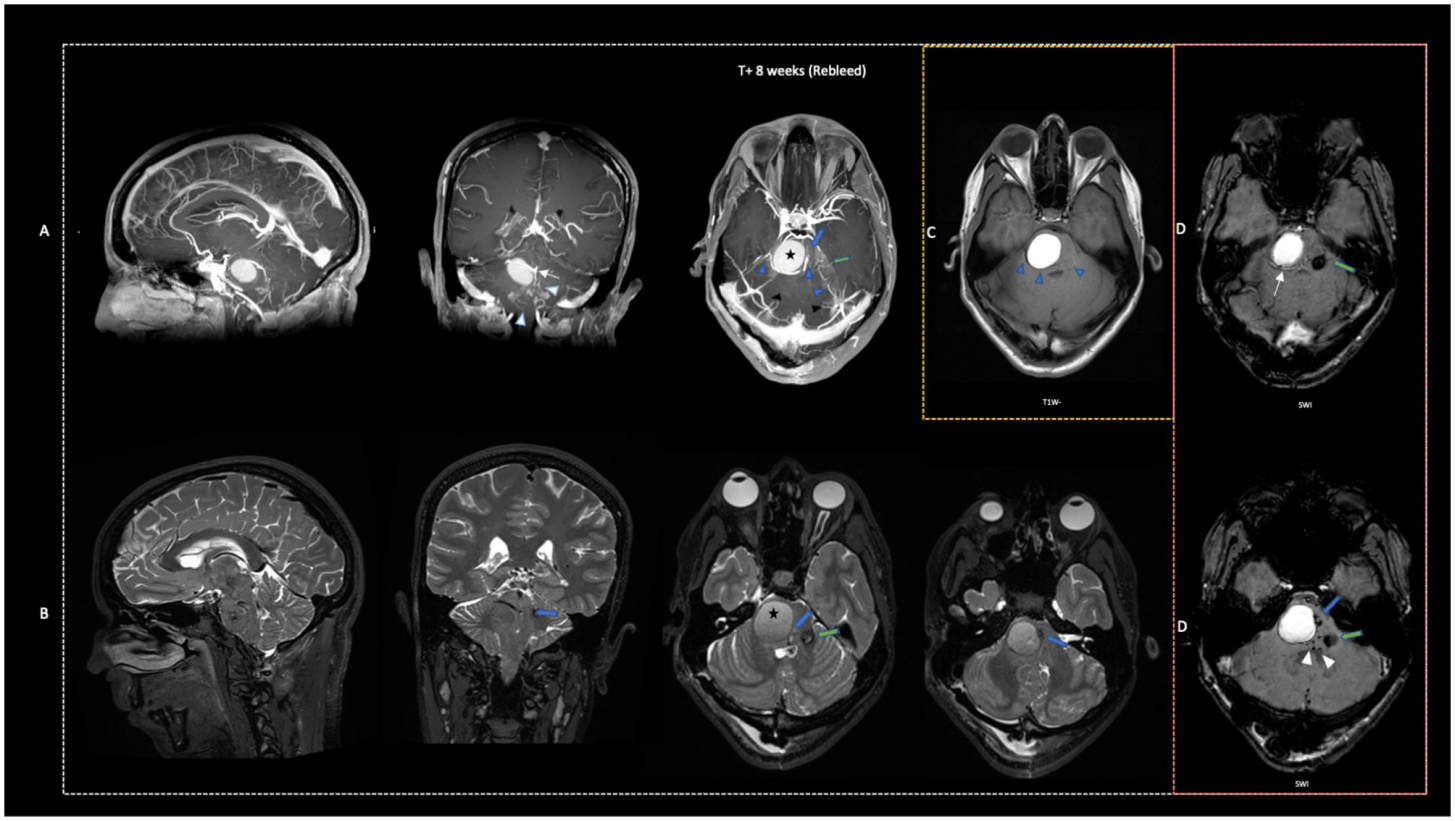
Rebleed after the first surgery. Sagittal, coronal, and axial MRI: **(A)** T1W + Gad; **(B)** T2W; axial only: **(C)** TW1 and **(D)** SWI. Showing late-stage subacute hematoma or extracellular meth-Hb (black star) with uniform hyperintense T1W signal and iso-hyperintense T2W signal. Target CCM (blue arrow) and untreated contralateral CCM (green arrow) seen as mixed signal intensity on T2W and blooming artifacts on SWI remain. Uniform filling of the main DVA draining vein but slightly stretched over hematoma as it drains into collecting vein on T1W+ (white arrow) and SWI (white arrow). Evidence of elevated pressure in the DVA system from the recurrence of prominent connecting channels (white arrowheads) on T1W+ and SWI.

The patient recovered from the first surgery with no new neurological deficits, and a satisfactory post-operative MRI showed an improved caliber of the DVA with reduced prominence of the connecting venous channels (Supplementary Figure 3). Further surgery to resect the target cavernoma was scheduled after 1 month. Unfortunately, the patient tested positive for the SARS-CoV-2 virus and re-presented 21 days later with hemiparesis (grades 3–4). MRI confirmed further hemorrhage in the surgical cavity, and the DVA continued to fill on gadolinium-enhanced T1W MRI, appearing stretched over the cavernoma with the reappearance of the prominent connecting channels (Figure 3).

Further surgery to resect the cavernoma was performed. The position of the long tracts and craniotomy relative to the lesion is seen in Supplementary Figure 4. Telovelar dissection performed at the end of the first surgery was extended to provide good exposure to the floor of the fourth ventricle. The right infrafacial EZ was approached through the sulcus limitans to completely resect the cavernoma and preserve the DVA. Neurophysiology mapping of the facial colliculus was performed as well as monitoring of the long tracts. A lesion consistent with a cavernoma was successfully removed at this surgery *en bloc* (see Supplementary Video).

Post-operative MRI confirmed complete resection of the cavernoma, preservation of the DVA, and discrete edema around the right facial nerve nucleus (Supplementary Figure 5). The patient suffered from a transient (7 days) post-operative right facial palsy (House–Brackmann grade 2), cranial nerve VI with nystagmus on right gaze, IX and X weakness, nystagmus, and left hemiparesis (grade 4) with reduced sensation.

**Figure 4:**
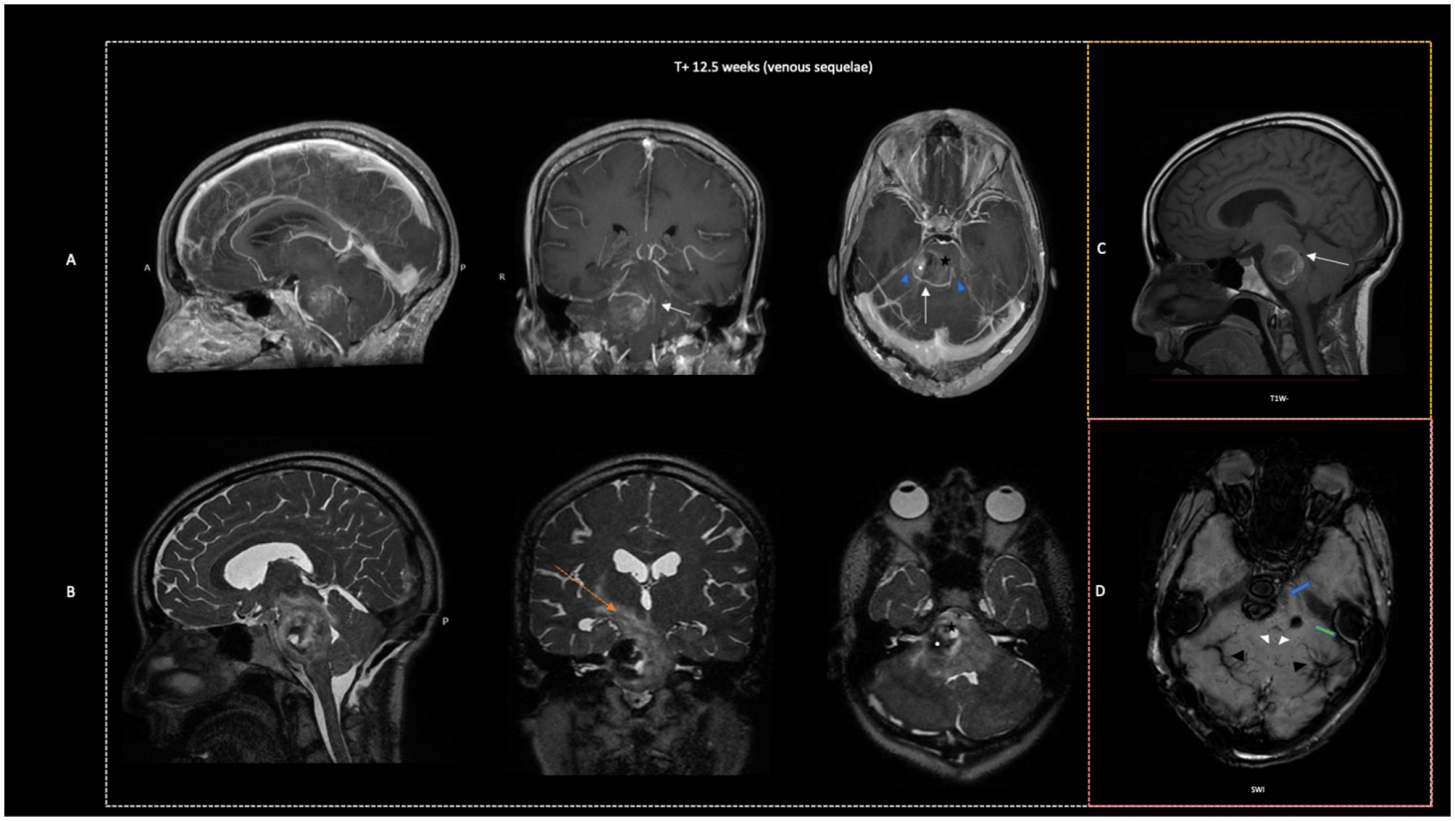
Delayed venous congestion and hemorrhage after the second surgery. Sagittal, coronal, and axial MRI: **(A)** T1W + Gad; **(B)** T2W; axial only: **(C)** TW1 and **(D)** SWI. Marked edema extending from the pons, middle cerebellar peduncles to the cerebral peduncles and subthalamus (orange arrow) on T2W. Reduction in size of the surgical cavity with a small volume acute hematoma (black star) seen as T1W hyperintense and T2W hypointense signal, and early subacute hematoma (white star) with T1W+ isointense and T2W hyperintense signal. The target CCM (blue arrow) appears to have been resected with no blooming artifact at this location seen on SWI. The contralateral CCM remains (green arrow) on SWI. Reduced filling caliber of main DVA draining vein on T1W+ (white arrow) with outline present but no hypointense signal on SWI. Difficult to comment on the pressure of the DVA system in the presence of marked edema.

Eighteen days after complete resection the complete resection of the cavernoma, the patient developed new headaches, incoordination, deterioration to grade 3 power, and a right abducens nerve palsy. Repeat imaging revealed delayed, extensive hyperintense T2W changes in the pons, middle cerebellar peduncle, and cerebral peduncle in keeping with venous infarction or congestion. This was associated with a small-volume acute/subacute hemorrhage in the surgical cavity and parenchyma, with a reduction in the caliber of the DVA and its collector vein on TW1+ (Figure 4).

**Figure 5:**
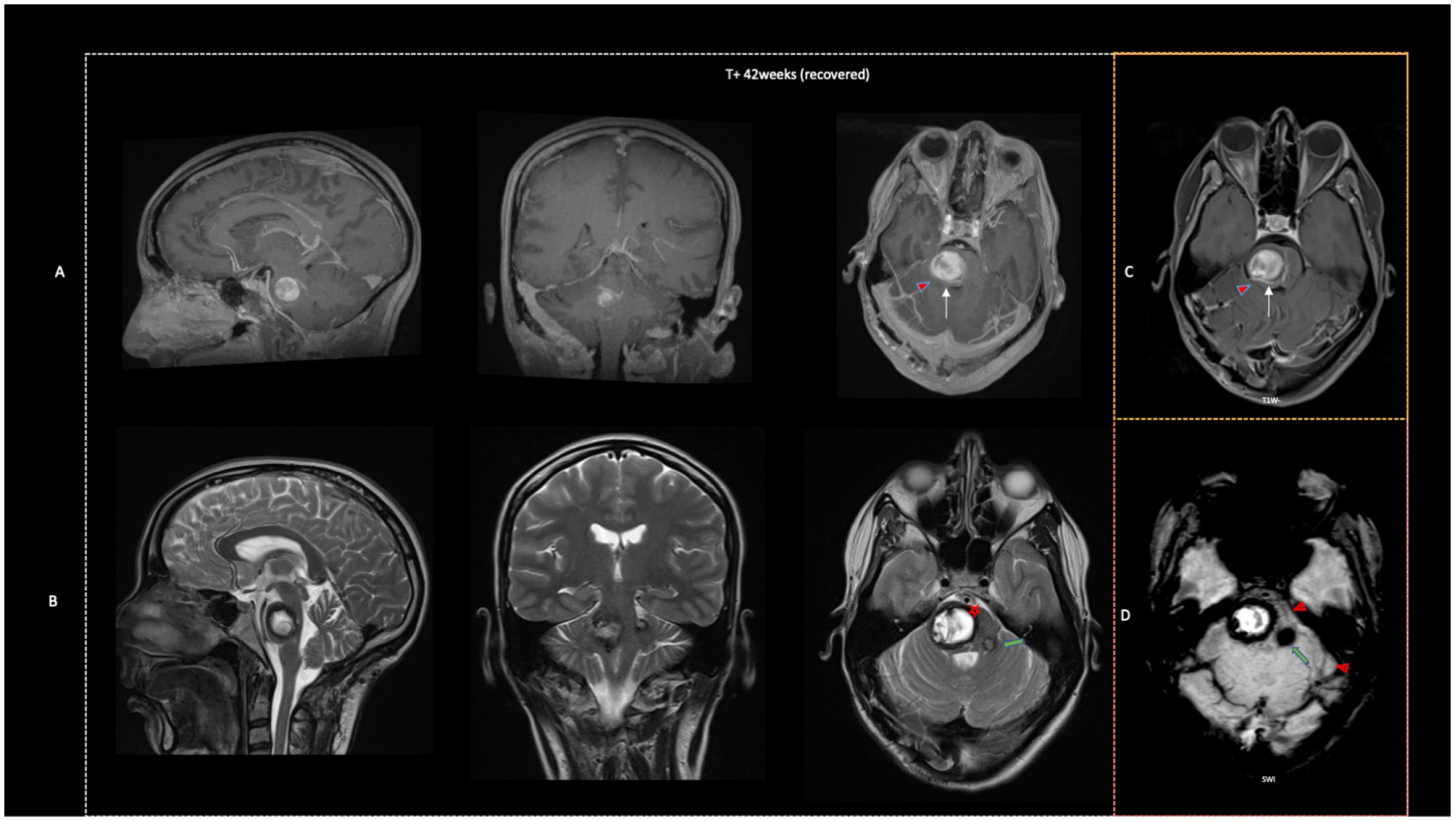
Improvement at 6 months. Sagittal, coronal, and axial MRI: **(A)** T1W + Gad; **(B)** T2W axial only: **(C)** T1W; and **(D)** SWI. Signal characteristics consistent with late-stage subacute hematoma or extracellular meth-Hb are seen as persisting T1W and T2W hyperintense signals (black star) in the surgical cavity. The additional appearance of a thick hemosiderin ring with hypointense T1, hypointense T2 signal (red star), and clear evidence of thrombus (T1W hyperintensity with reduced filling on T1W + gad) within the DVA system on the right (red arrowhead). The target CCM is confirmed as no longer present on T2* and SWI. The contralateral CCM (green arrow) remains.

The picture was interpretted as venous hemorrhagic infarction secondary to outflow restriction of the main draining vein of the DVA. The patient was managed with a tapering course of dexamethasone (16 mg daily) with improvement in the edema (Supplementary Figure 6) and limb weakness (grade 4) but unchanged cranial nerve deficit after 2 weeks. MRI at discharge showed an asymptomatic late-subacute stage hematoma in the cavity with the return of prominent connecting venous channels and uncompressed caliber of the main draining vein of the DVA. A further MRI, performed 6 months following the last surgery, confirmed no residual targeted CCM, thrombosis within the DVA draining vein, and persistence of the contralateral untreated CCM associated with an increase in prominence of its related anomalous veins (Figure 5). The patient was independently mobile and able to attend to her activities of daily living but not back to work at this review. Abducens palsy and hemiparesis had achieved near-complete recovery.

The present case discusses the nuances of management and provides insight into common (hemorrhage) and rare (delayed venous) complications of BSCM surgery.

## 3. Discussion

Microsurgery in experienced centers is an accepted strategy for the management of brainstem cavernous with compression hematoma or mass effect. Despite the advances in pre-operative planning with tractography, the use of “safe” microsurgical entry zones, and neurophysiological monitoring during surgery, morbidity cannot always be averted. A large meta-analysis of outcomes following surgery showed ~ 90% of cases achieved complete resection, with residual CCMs re-hemorrhaging in two-thirds of cases (19). One-third of cases experienced postoperative morbidity, with cranial nerve function more likely than limb function to be affected, but most patients had recovered by the final clinical review (19). Respecting nearby intramedullary veins is generally accepted by expert consensus to be important to avoid complications in brainstem cavernoma surgery (16). The present case shows that this complication may develop in a delayed manner, even when the DVA is preserved.

### 3.1. Anatomy, histology, and hemorrhagic risk

Cavernous malformations appear to be more frequently intimately related to DVA or prominent medullary veins than is reported on standard pre-operative imaging (10, 11). Understanding the relationship between these veins, their collector vein, and the cavernoma is important to preserve them during surgery. Lasjaunias et al. identified five patterns of transmedullary venous drainage in the posterior fossa: drainage into the precentral vein, vermian veins, cerebellar veins, petrosal vein confluence, and transpontine vein (16). DVA drainage into the petrosal vein confluence and veins of the lateral recess appears to be the most frequently encountered pattern (20, 21).

Dammann et al. (11) confirmed this by showing that single or multiple venous outflow vessels were present in most cases of CCMs on SWI on 7T MR imaging. The classic pattern of caput medusae appearance with a dilated collector vein was observed in 30% of cases. The group identified two other consistent variants in their study: type 2 with a solitary transcerebral or subpial draining vein (observed in 18% of cases), and type 3 comprised of multiple transcerebral veins from the CCM in a reticular structure that drained adjacent brain tissue (in 52% of CCMs) (11). They identified that all venous malformations were connected either to the superficial or deep parapontine draining system (11). The DVA in our patient was closer to a type 3 variant and drained into the superior petrosal sinus *via* the petrosal vein confluence.

The absence of a clear pattern of drainage in the prior series reflects the resolution of different MR sequences and that of SWI at low field strengths. The classic DVA with a direct connection to dilated transcortical veins is more readily observed on lower-resolution MR sequences than those with undilated collecting veins (22).

Hypertension in this collection system may trigger hemorrhage, as suspected in the present case, at presentation and later following surgery. It may also result in delayed congestive edema of the pons and cerebellum following progressive outflow restriction post-operatively. Small connections between the DVA, both cavernomas and the hematoma noted in the present case on pre-operative gadolinium-enhanced T1W imaging, support the theory of “hemorrhagic angiogenic proliferation” described by Awad et al. as the pathophysiology of CCMs (22, 23). Sasaki et al. reported on a similar observation of several small veins connecting CCMs with the DVA seen intraoperatively (24). The physiological connection between the CCM and the venous circulation confirmed by Little et al. (25) during measurements of intraoperative cortical blood flow may explain the hemorrhagic sequela from DVAs including after surgery.

### 3.2. Timing of surgery

The timing of microsurgical intervention for brainstem cavernomas is another aspect of management that can be chosen to limit surgical morbidity. Surgery during the subacute phase (4–6 weeks) following an index hemorrhage is reported to reduce surgery-related trauma as the liquefaction of the hematoma demarcates cavernoma from eloquent tissue (16, 26). Prolonged delay risks further hemorrhage with the attendant concern of additional disability or death.

### 3.3. Anatomy of entry zones and DVA preservation

Tantongtip et al. (17) reported similar post-operative outcomes following the resection of a cavernoma regardless of whether the associated DVA was occluded or preserved. Despite this, most surgeons maintain the strategy of preserving these veins to be the safest (16). In our surgical video, we show how two brainstem entry zones can be used to reach the cavernoma while avoiding injury to the DVAs.

The peritrigeminal entry zone (EZ) that lies between cranial nerves V and VII can be reached through the retrosigmoid or far-lateral approach used in the first surgical attempt (18). The corticospinal tract in the absence of disease lies ~ 8.6 mm anterior to this EZ. To preserve the trigeminal, abducens, and facial nerve function, the craniocaudal limits of the corridor should not be extended (18, 27). The hematoma cavity displaced the tracts in the pons and limited injury to the vital structures. The trigeminospinal tract and medial and lateral lemnisci lie at the posteromedial depth of the corridor. Visualization of the cavernoma which was in the medial aspect of the cavity from this entry zone was limited.

The sulcus limitans was used as a landmark in the fourth ventricle floor during the second surgery to avoid the medially located DVA. The infrafacial EZ offered a good working corridor in the rostro-caudal axis of about 12.6 mm (18). We limit extension toward the midline to avoid injury to the middle longitudinal fasciculus and laterally to preserve the central tegmental tract (18, 27). The nuclei of cranial nerves VII and VI must be avoided. The medullary stria and lateral recess serve as useful landmarks for this (18). This access corridor may have predisposed to the facial and abducens palsies suffered by the patient after venous ischemia.

Although we performed an exposure in the first case to provide access to both entry zones, including telovelar dissection, the peritrigeminal EZ was chosen as the first approach because it avoided manipulation of the DVA and cranial nerve nuclei deep to the floor of the fourth ventricle. The disadvantages were that while it allowed access to the hematoma cavity, direct visualization without the aid of an endoscope and specialized instruments was limited. Approaches through the floor of the fourth ventricle including the transmedian sulcus, and infra- and suprafacial corridors provide direct visualization and access to the cavernoma but at a higher risk of injury to the displaced nuclei and tracts. Following drainage of the liquid hematoma and deformation of the cavity in the first surgery, we opted against using the latter at the same sitting because the risk of injury was felt to be higher without additional imaging to localize the cavernoma.

### 3.4. Risk factors for spontaneous DVA occlusion

Spontaneous occlusion of DVA is a rare event, with few reported cases showing that this may lead to venous infarction or hemorrhage (13).

The lack of a smooth muscle layer and elastic lamina in DVAs may limit their ability to regulate flow and predispose them to thrombosis (28, 29). This may also contribute to the latent venous hypertension in DVAs which has been suggested by some authors to predispose to cavernoma formation and hemorrhage (30). Radiological “occult” or atypical DVAs have been histologically shown to comprise venous channels without smooth muscles, while MRI apparent DVAs have dilated thin-walled vessels diffusely distributed in the normal white matter (28, 30). The changes in the caliber and prominence of the venous channels observed in the present case are likely to be within the limits of the scanner but are worth further assessment in a larger prospective series.

Cerebral cortical vein thrombosis and DVA thrombosis have been postulated to have similar risk factors (13). COVID-19 infection or vaccination in the last 30 days is a recognized cause of hypercoagulability, which increases the risk of cortical venous thrombosis (31). COVID-19 infection following surgery may have contributed to the DVA-related complications observed in the present case.

We hypothesize that a combination of intrinsic hypercoagulability, stretching or compressing the DVA by the post-operative cavity, and manipulation during surgery may have contributed to delayed venous outflow with venous congestion and hemorrhage as sequelae. The small volume of hemorrhage relative to the venous edema makes it more likely the hemorrhage resulted from rather than caused the outflow obstruction.

### 3.5. Management

Anticoagulation has been suggested for the management of symptomatic DVA thrombosis (13, 32), given similarities with CVT where it is recommended even in the presence of hemorrhage (33). Expansion of a brainstem hematoma is likely to be fatal, with microsurgical management of BSCMs associated with hemorrhage anecdotally considered safer regardless of etiology. However, the successful use of anticoagulation in the literature (13) for non-hemorrhagic sequelae suggests cautious use in the weeks following surgery may prevent delayed thrombosis. In the present case, thrombosis within the DVA became more radiologically apparent 6 months following surgery. Although the role of thrombosis in inducing CCM hemorrhage remains unclear, a large cohort study and meta-analysis found the use of antithrombotic therapy to be associated with a lower risk of intracranial hemorrhage in patients with a CCM (34).

More importantly, we recommend judicious hemostasis to limit small volume operative site hematoma that may evolve to cause delayed mechanical obstruction of the DVA.

We acknowledge the use of corticosteroids in cerebral venous thrombosis is controversial, including reports by Canhão et al. (35) of worse outcomes in patients. The rationale for our use of corticosteroids for brainstem edema was driven by the possible COVID-related mechanism for the DVA outflow restriction or thrombosis after the first surgery, and the associated mass effect of the hematoma. Corticosteroid therapy is effective in reducing vasogenic edema, although no large evidence base exists to support its use following venous injury. Venous congestion, as in the present case, results in both cytogenic and vasogenic edema with the latter likely to respond to steroids. The clinical and radiological observation of our patient adds to the literature for its use in DVA thrombosis.

### 3.6. Limitations

We present only one case to support our observations which is an inherent limitation of a case study. An endoscope may have aided visualization of the cavity in the first surgery; however, the impact of adjuncts to surgery is beyond the scope of the study.

### 3.7. Patient perspective

I recognized this complication could have occurred without the surgical intervention I opted for and remain of the view that surgery was a reasonable choice given the progressive deterioration at the start of my journey.

## 4. Conclusion

Knowledge of brainstem corridors is invaluable to planning microsurgery for brainstem cavernomas. The associated risks of injury to nearby DVA extend beyond the operating room. Understanding how to investigate and manage this complication is an important aspect of cavernoma surgery.

The present case of delayed DVA outflow obstruction, causing cerebellar and pontine venous congestive edema, after surgical resection of a brainstem cavernoma is extremely rare. Careful inspection of imaging for an associated DVA, and detailed perioperative planning including strict hemostasis may be key to avoiding this rare complication of brainstem cavernoma surgery.

## Data availability statement

The raw data supporting the conclusions of this article will be made available by the authors, without undue reservation.

## Ethics statement

Written informed consent was obtained from the individual(s) for the publication of any potentially identifiable images or data included in this article.

## Author contributions

KA and RG: preparation, editing of video, illustration, and manuscript. SM and AF: review and editing of the manuscript. VR: a review of radiology images and manuscript preparation. All authors contributed to the article and approved the submitted version.
