# Supplementary material for: Case report: Delayed outflow obstruction of a DVA: A rare complication of brainstem cavernoma surgery
Source: Front Neurol. 2023 Mar 14;14:1073366. doi: 10.3389/fneur.2023.1073366 (PMC10044343; doi:10.3389/fneur.2023.1073366)
Supplement: Supplementary file 2 [file Data_Sheet_1.docx]

Supplementary Material


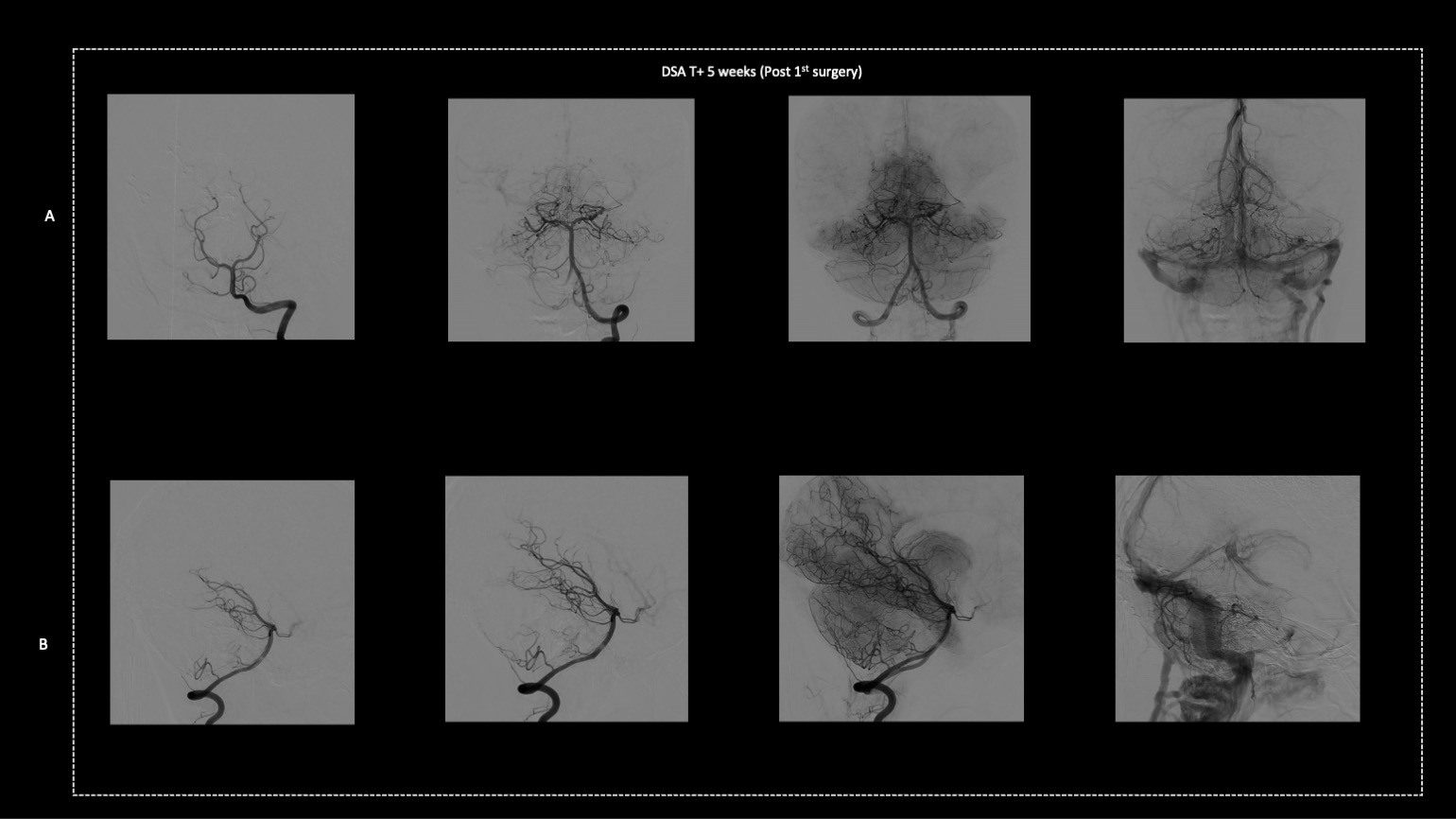


**Supp 1**

**
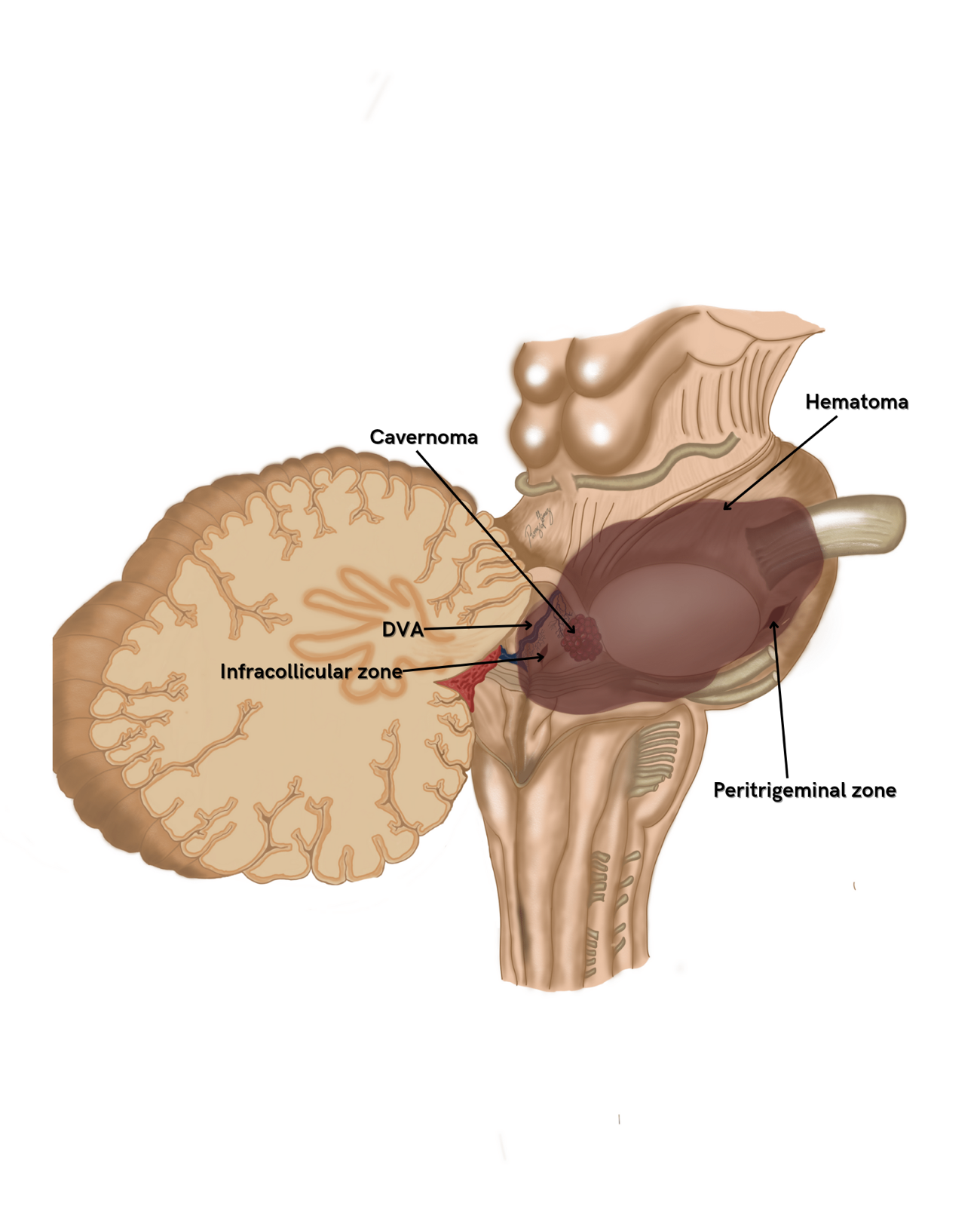
**

**Supp 2A**


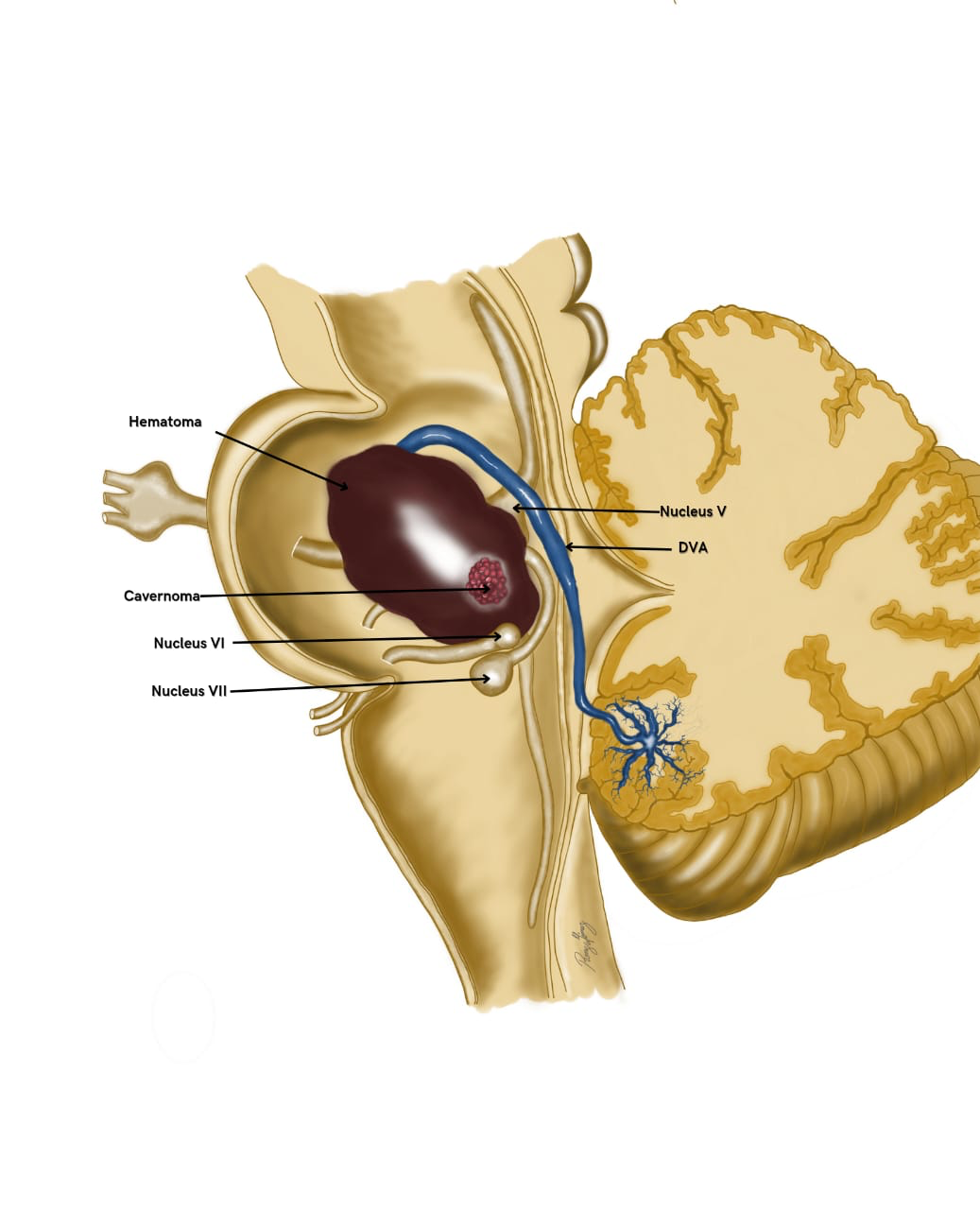


**Supp 2B**


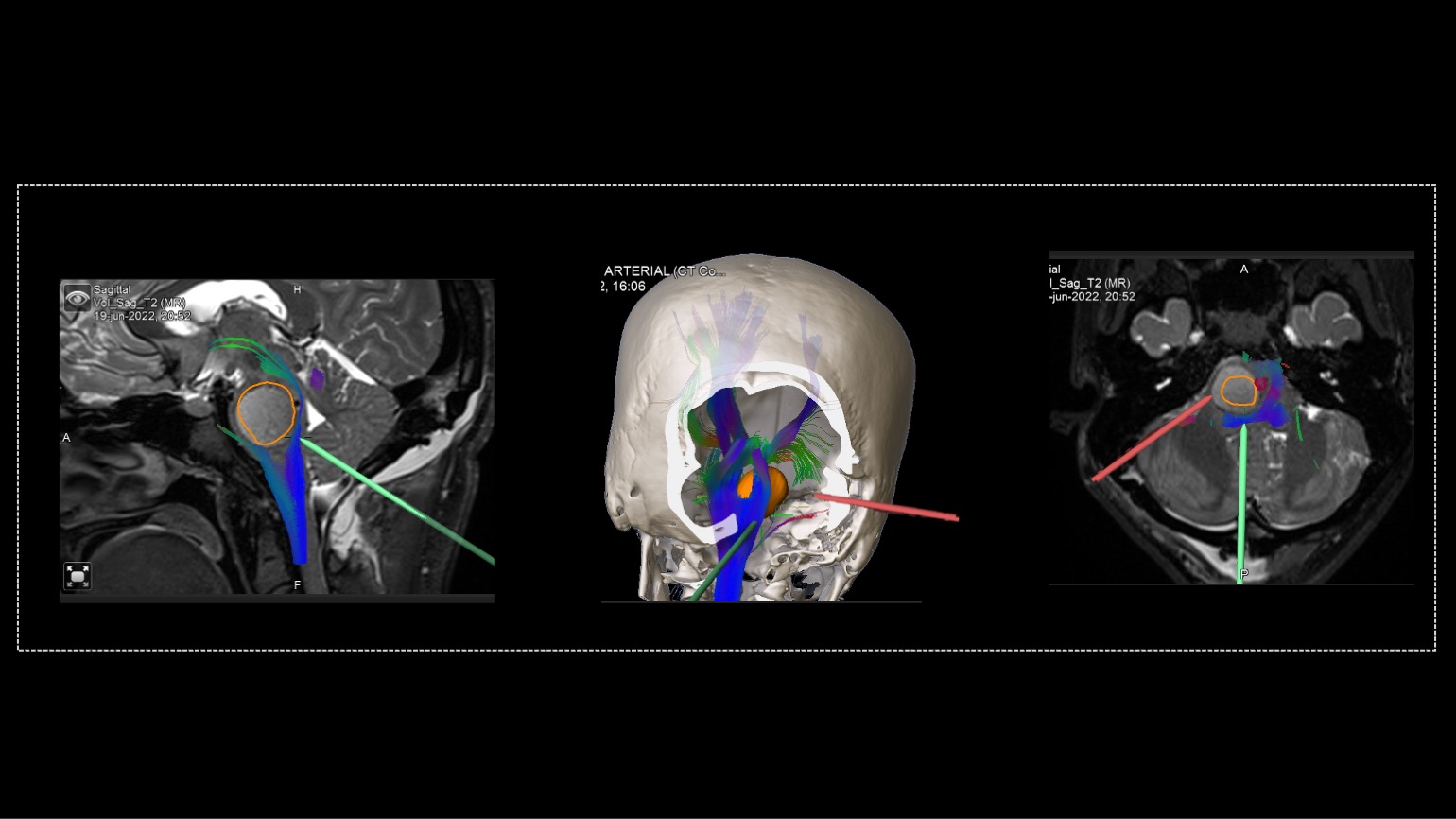
**Supp 3**

**
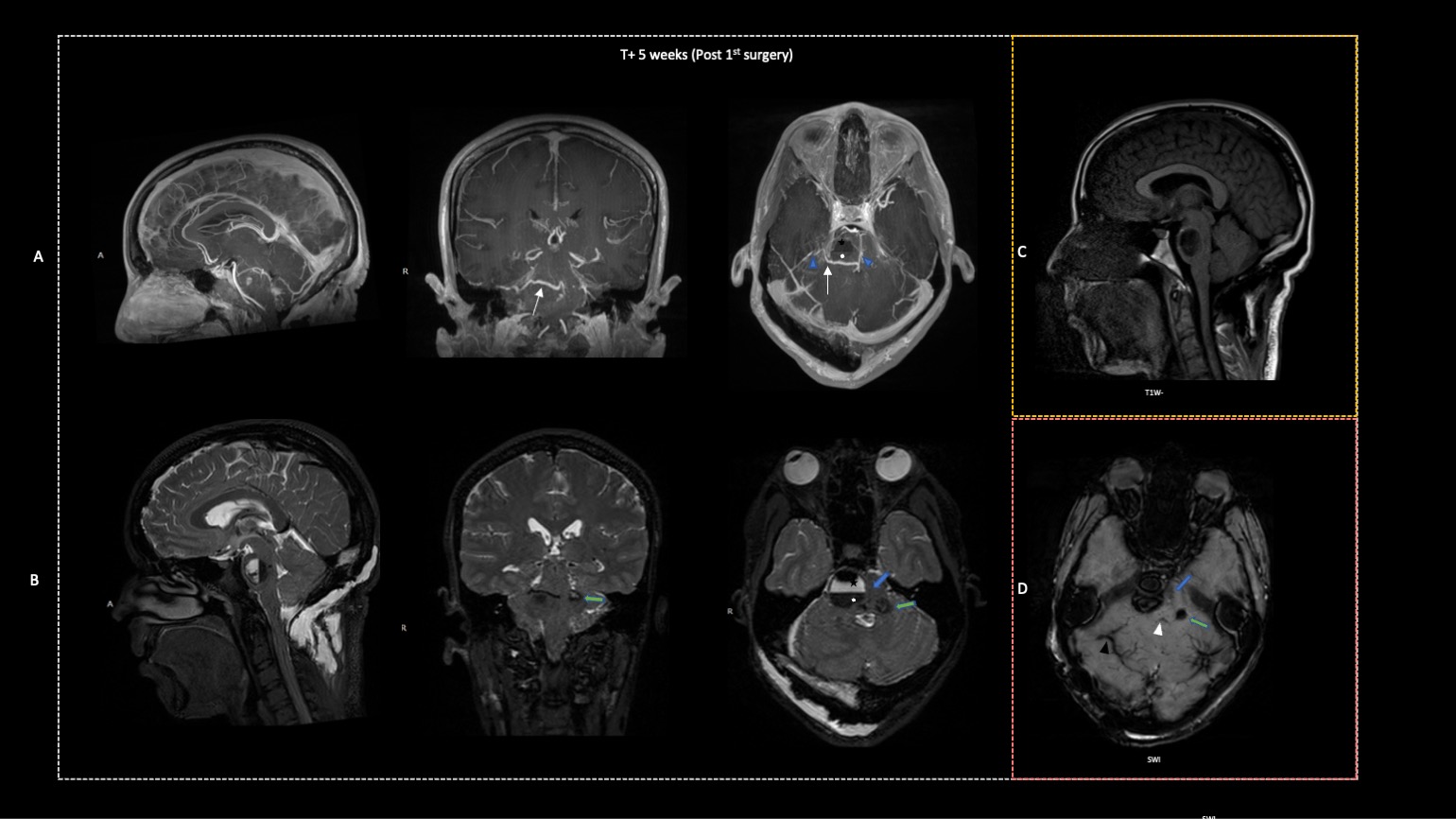
Supp 4**

**
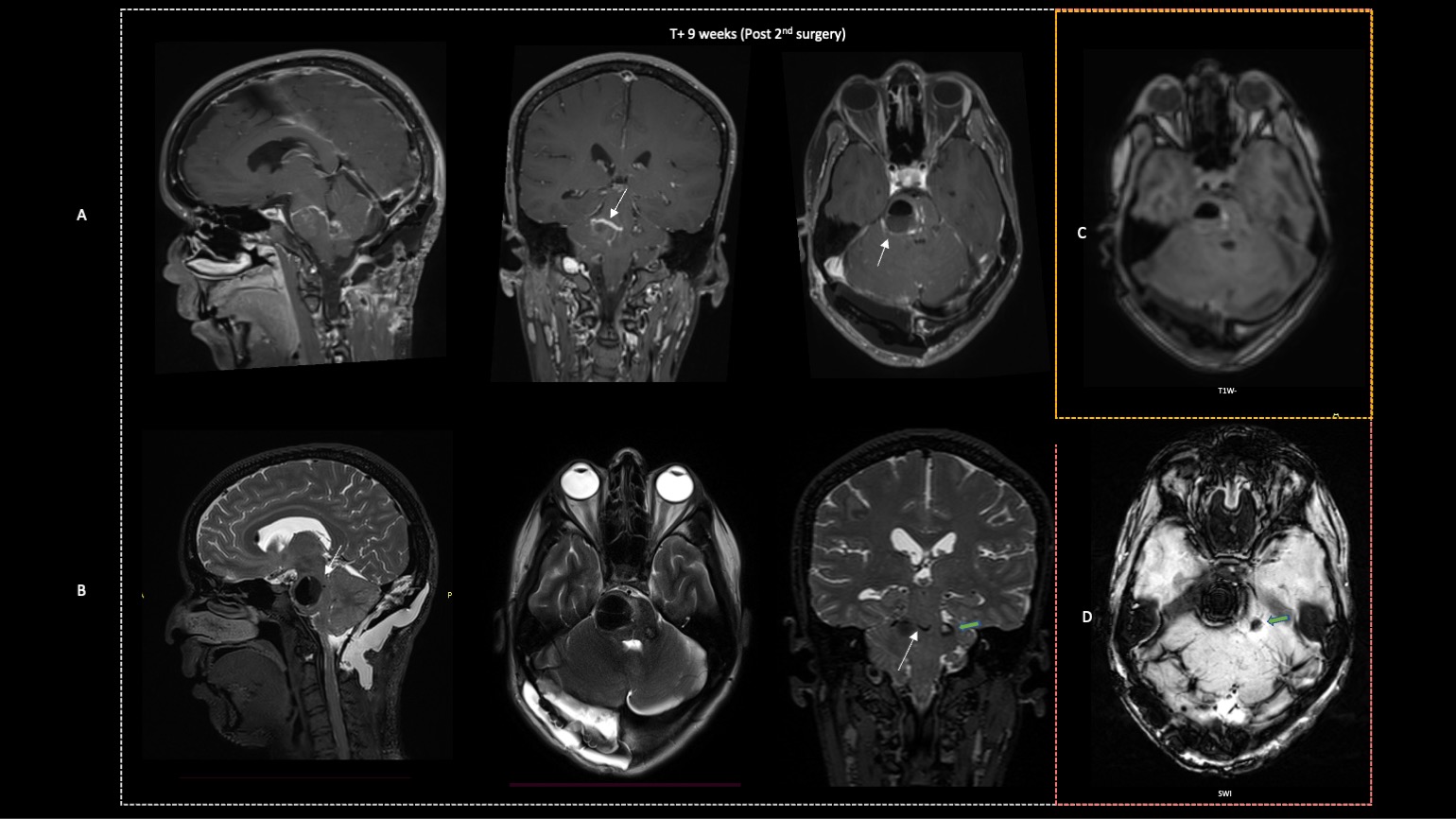
Supp 5**


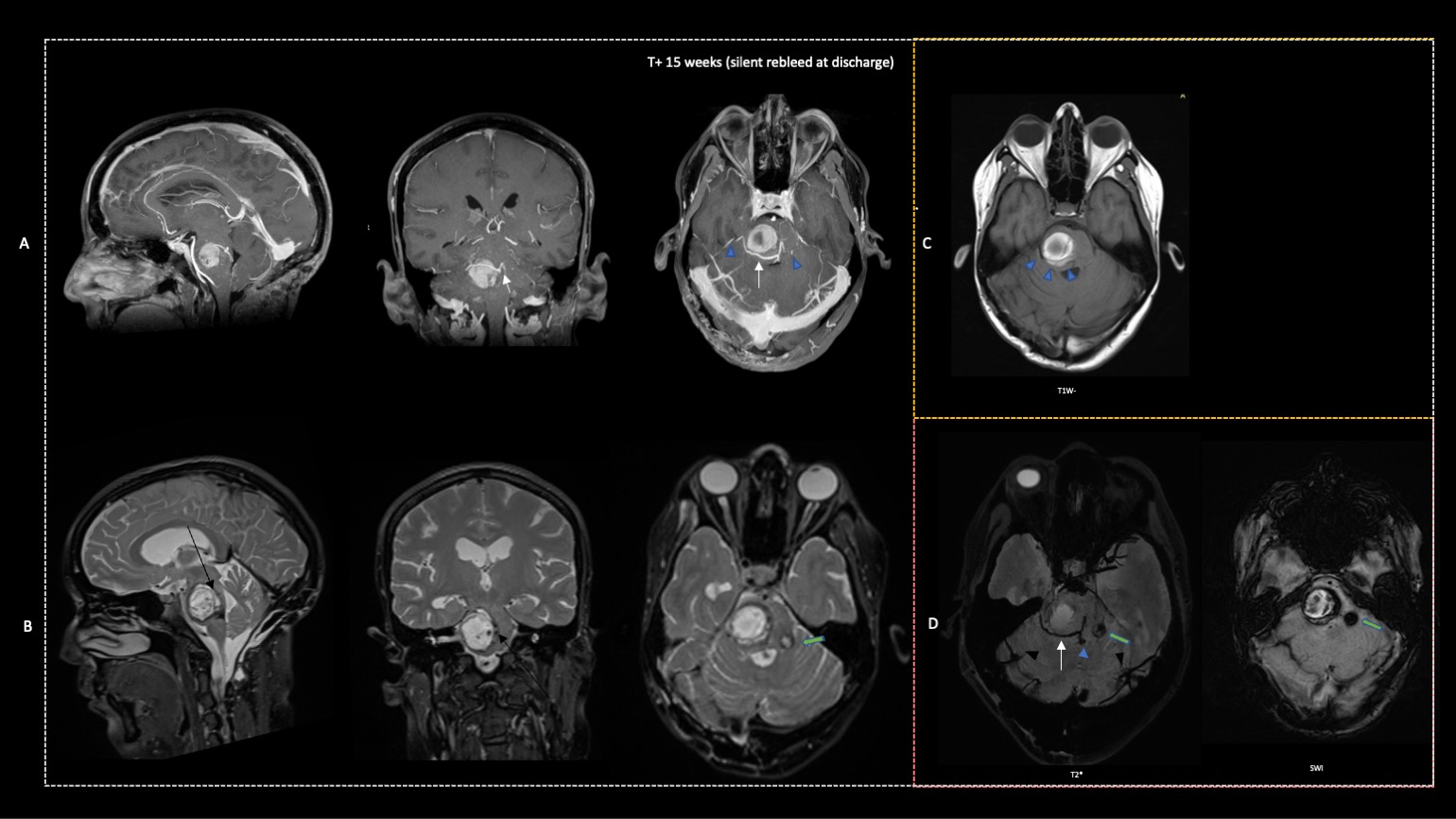


**Supp 6**

**Supplementary material legend**

**Supp 1. A) Antero-posterior and B) lateral view of DSA, vertebral injection**

No arteriovenous malformation, fistulae, or aneurysm. DVA visualized in venous phase.

**Supp 2. Illustration of brainstem with pontine hematoma, cavernoma, DVA and the two surgical entry zones used this case.**

**Supp 3. Combined MRI tractography with post-operative 3D CT reconstruction**

Tractography showing displacement of long tracts to the left, pontine hematoma, cavernoma and the two surgical entry zones used in this case. The trajectory of the retrosigmoid approach to peritrigeminal EZ (red arrow) and midline telovelar approach to infrafacial EZ (green arrow).

**Supp 4. Post-operative (1st surgery): Sagittal, coronal, and axial MRI: A) T1W+Gad; B) T2W; Axial only: C) T1W- and D) SWI.**

Showing a smaller post-operative cavity with signal features in keeping with recent surgery; air artefact on T1W, T2W and SWI.

Faintly visible target/culprit CCM (blue arrow) on SWI and blooming artefact of incidental contralateral cerebellar peduncle CCM (green arrow) on SWI with popcorn appearance of mixed T2W signal intensity.

Associated with

Increased Gadolinium filling caliber of the main DVA draining vein (white arrow) and other collector veins (blue arrowheads) seen on T1W+ compared to pre-surgery. T2W flow void also of improved caliber.

Pressure in the DVA system appears lower evidenced by reduced prominence of connecting channels (white arrowhead), less signal dropout in the collecting veins and caput medusae (Black arrowhead) on SWI.

**Supp 5. Post-operative (2nd surgery). Sagittal, coronal, and axial MRI: A) T1W+ Gad; B) T2W; Axial only: C) T1W- and D) SWI.**

Hypointense signal on T1W and T2W in the surgical cavity likely to reflect early post-operative changes of fluid and air. The target CCM appears resected on T2W and SWI. Improved caliber of draining veins of the DVA (white arrow) on T1W+ and T2W. Contralateral CCM (green arrow) remains. No evidence of venous infarction or significant surgical white matter changes or new hematoma.

**Supp 6. Clinically silent rebleed 6 weeks after 2nd surgery. Sagittal, coronal, and axial MRI: A) T1W+Gad; B) T2W; Axial only: C) T1W- and D) T2* and SWI.**

Enlarged cavity with late subacute or extracellular meth-Hb of T1W+ and T2W hyperintense signal (Black star). Appearance of new small volume parenchymal hematoma capping older encysted cavity (black arrow).

The target CCM is confirmed as no longer present on T2*, SWI. Contralateral CCM (green arrow) remains.

The main DVA appears to have an uncompressed caliber (white arrow) on T1W+, T2* but no outline seen on SWI

Likely low pressure within the DVA from fewer connecting channels on T1W+ and SWI

**Supplementary video:**

Showing the surgical approaches described, the DVA and removal of the cavernoma
